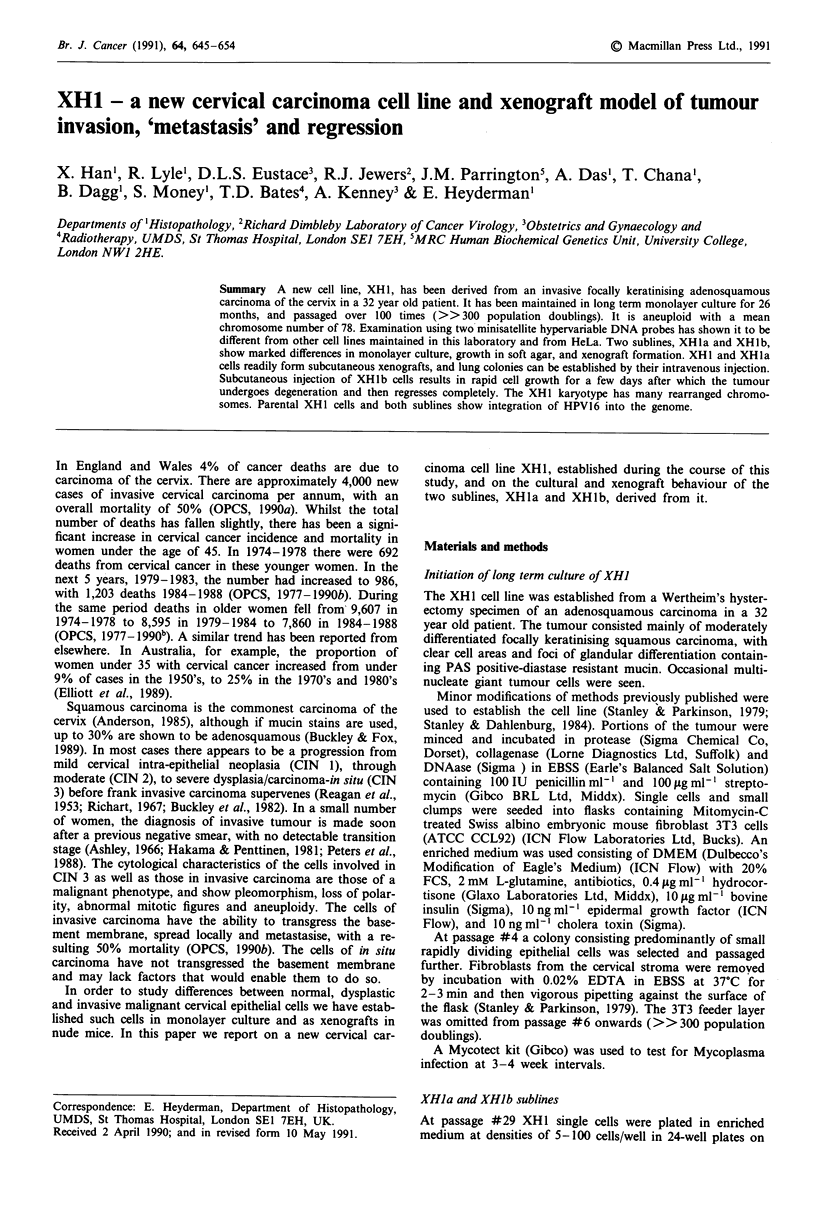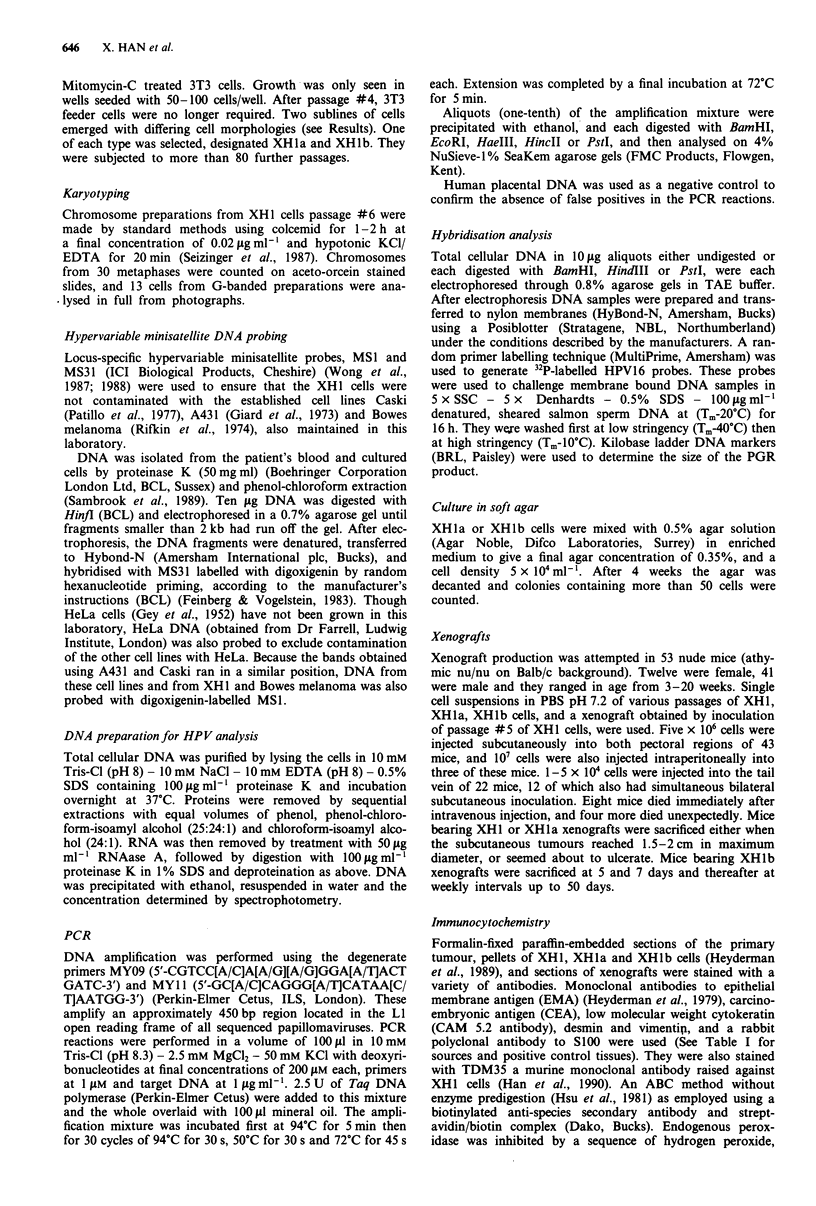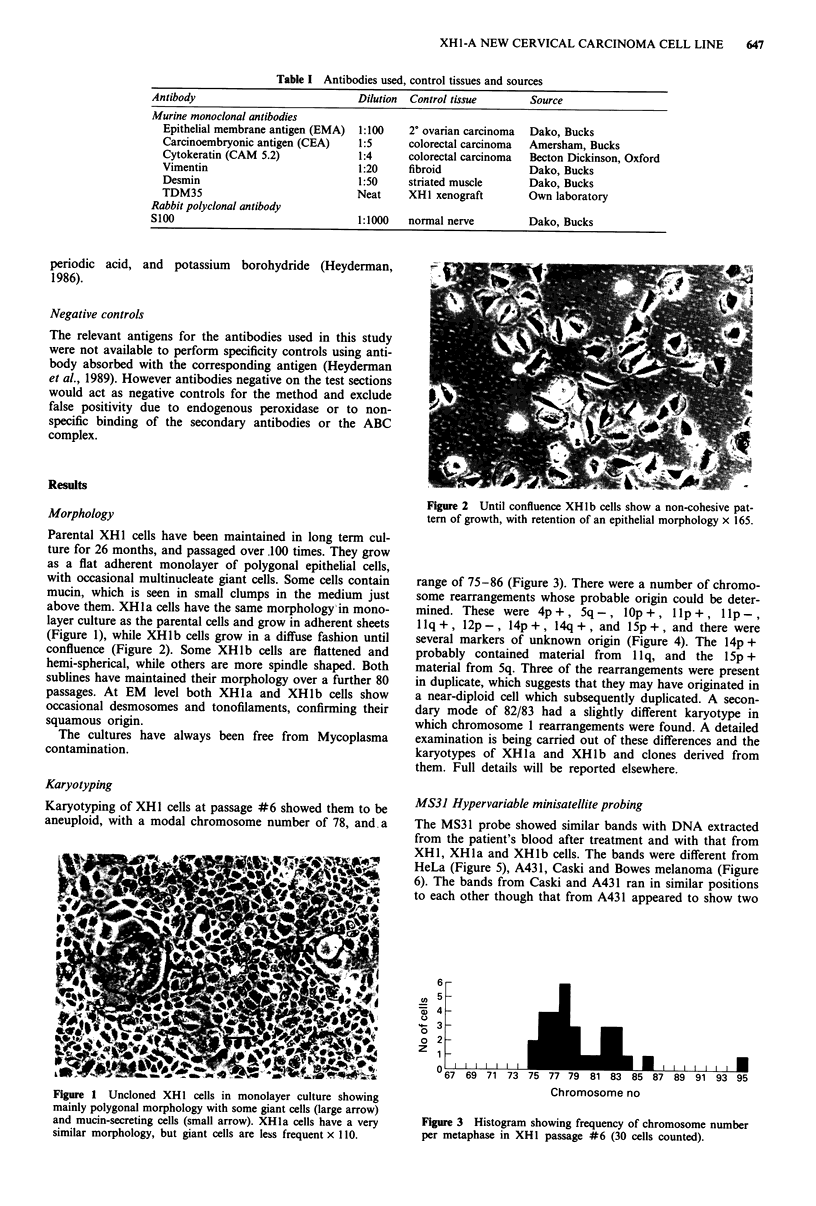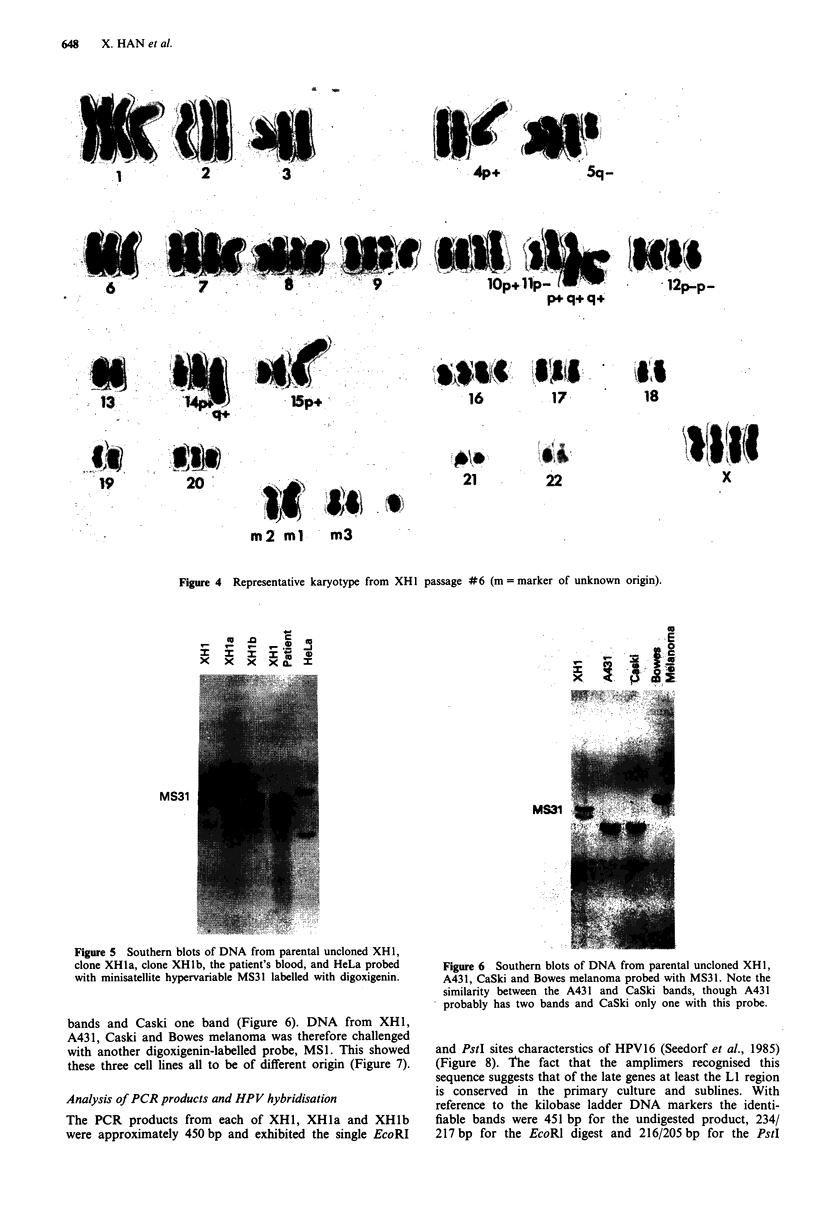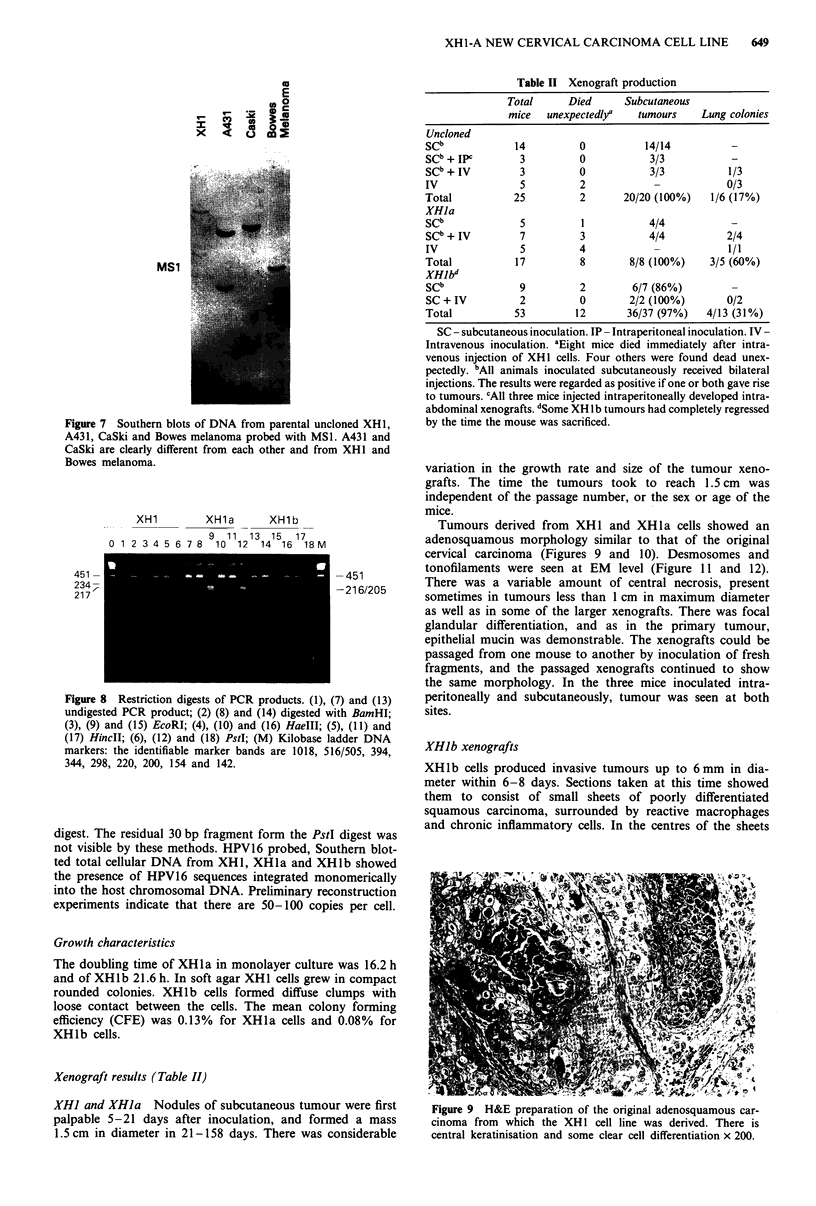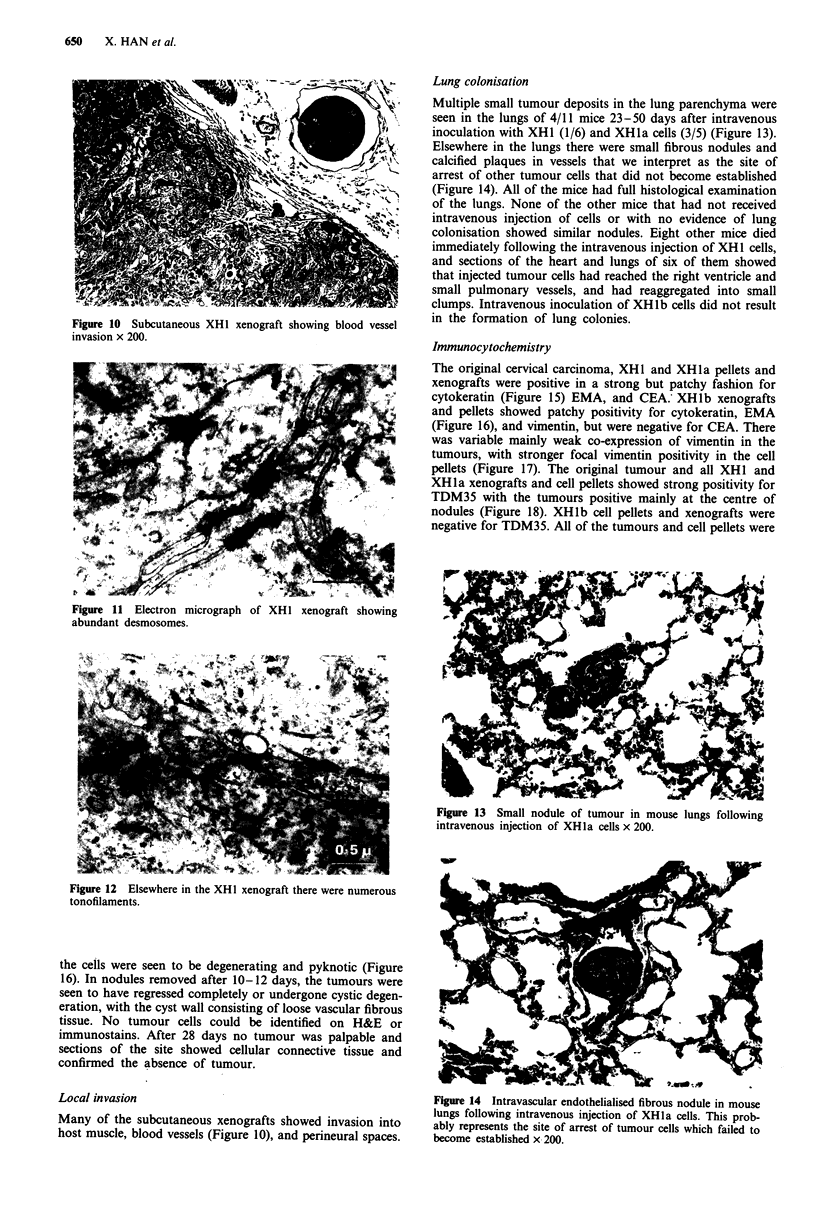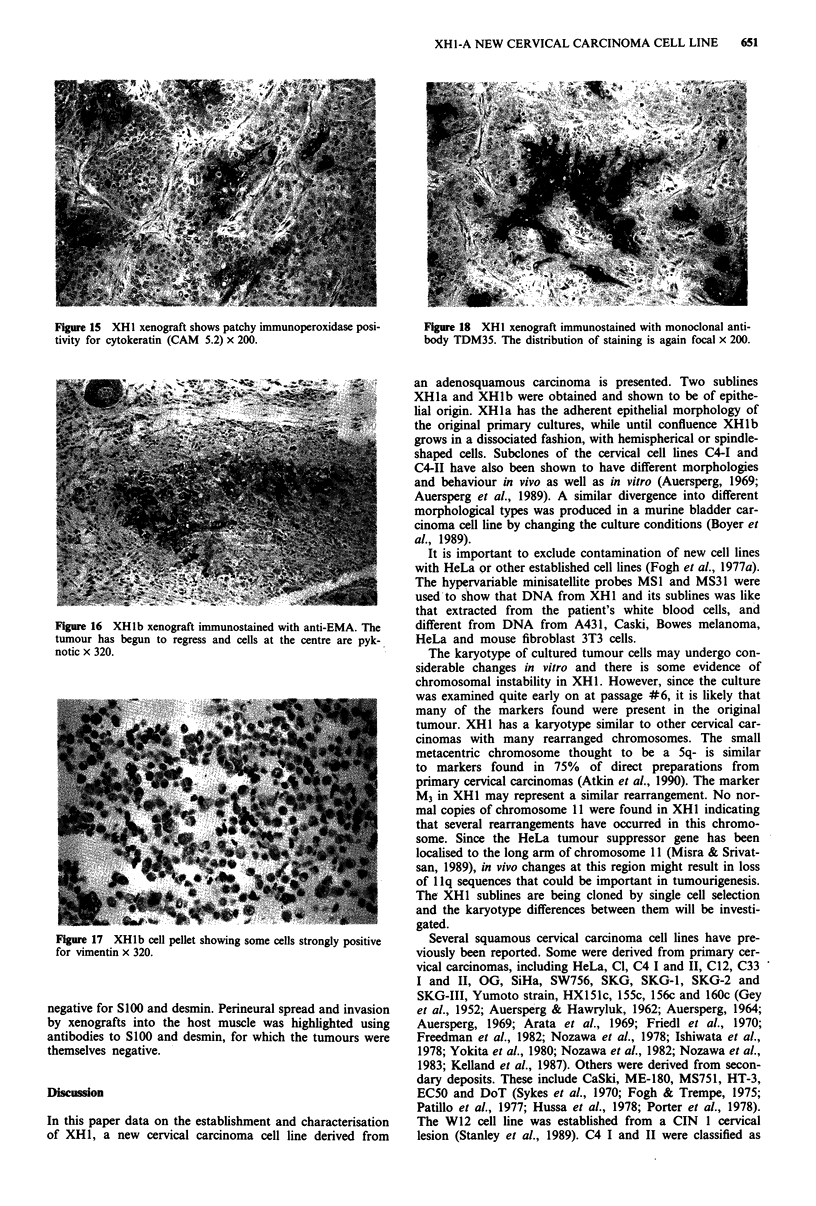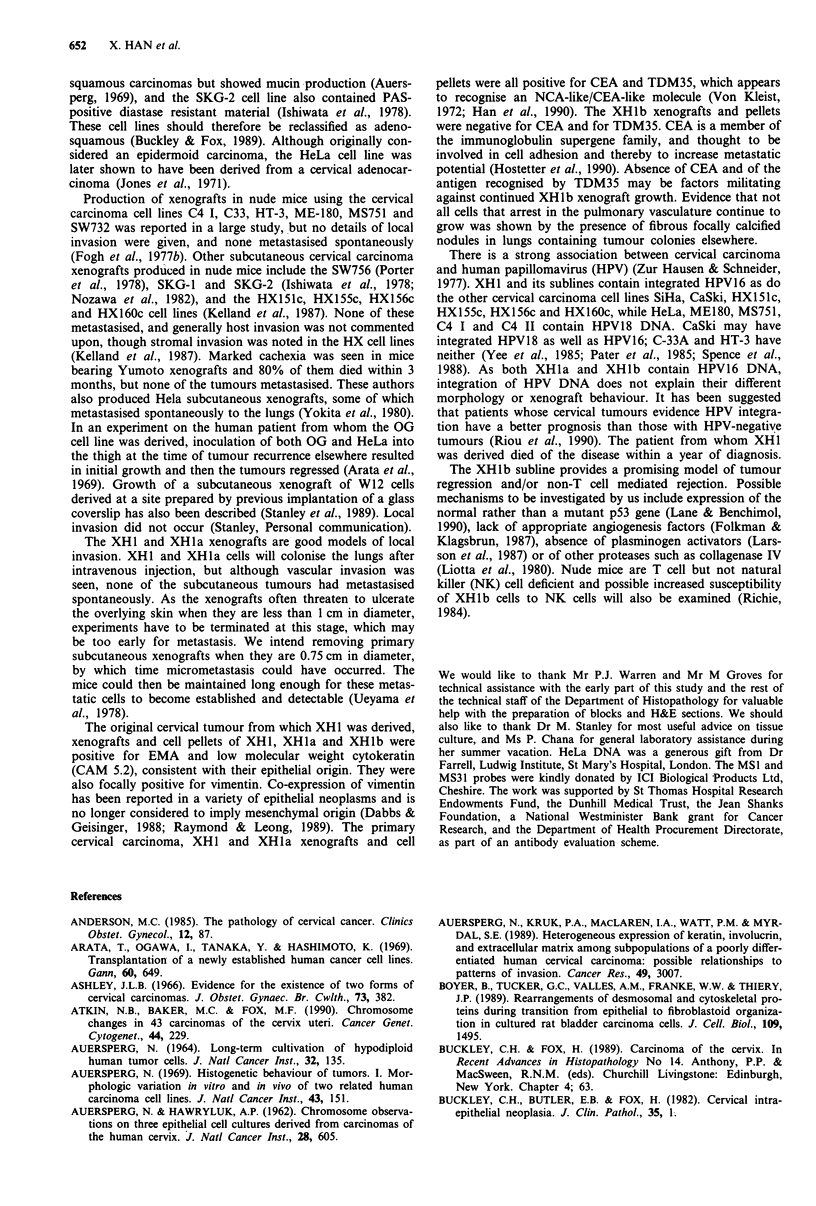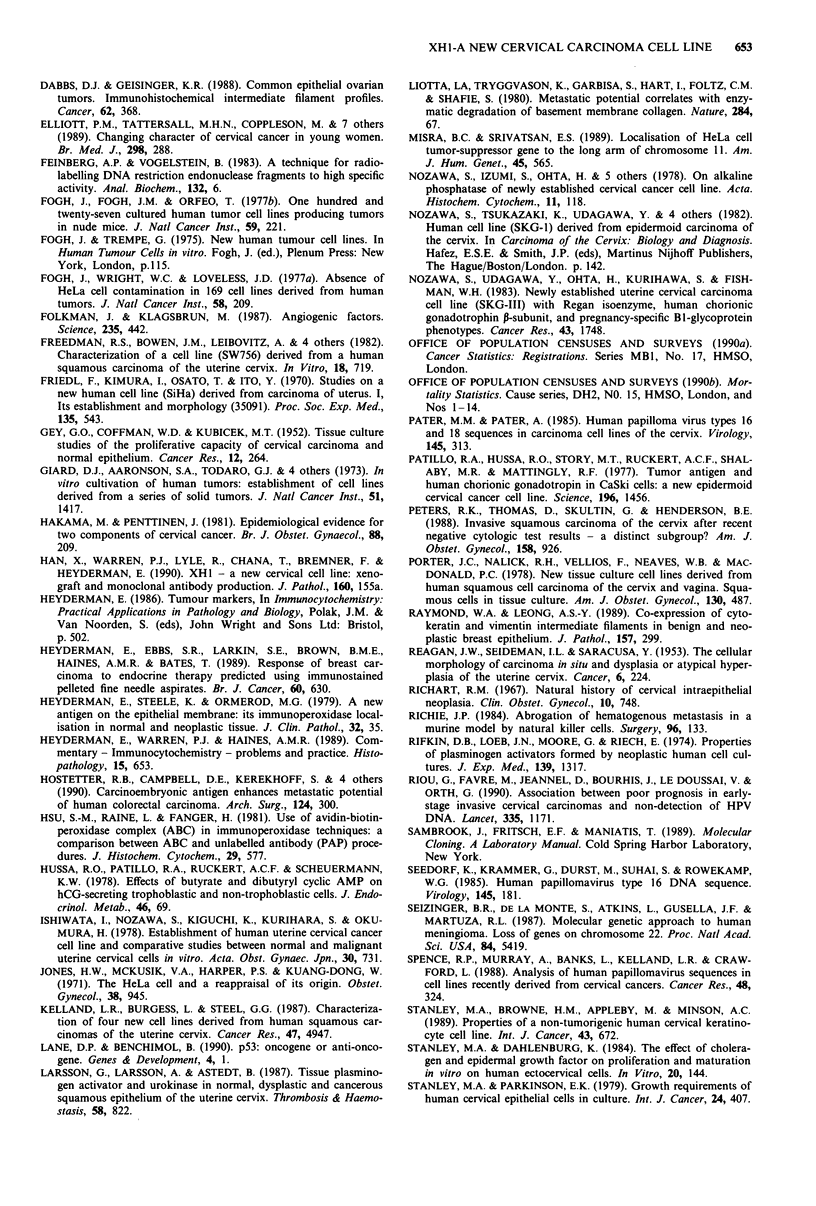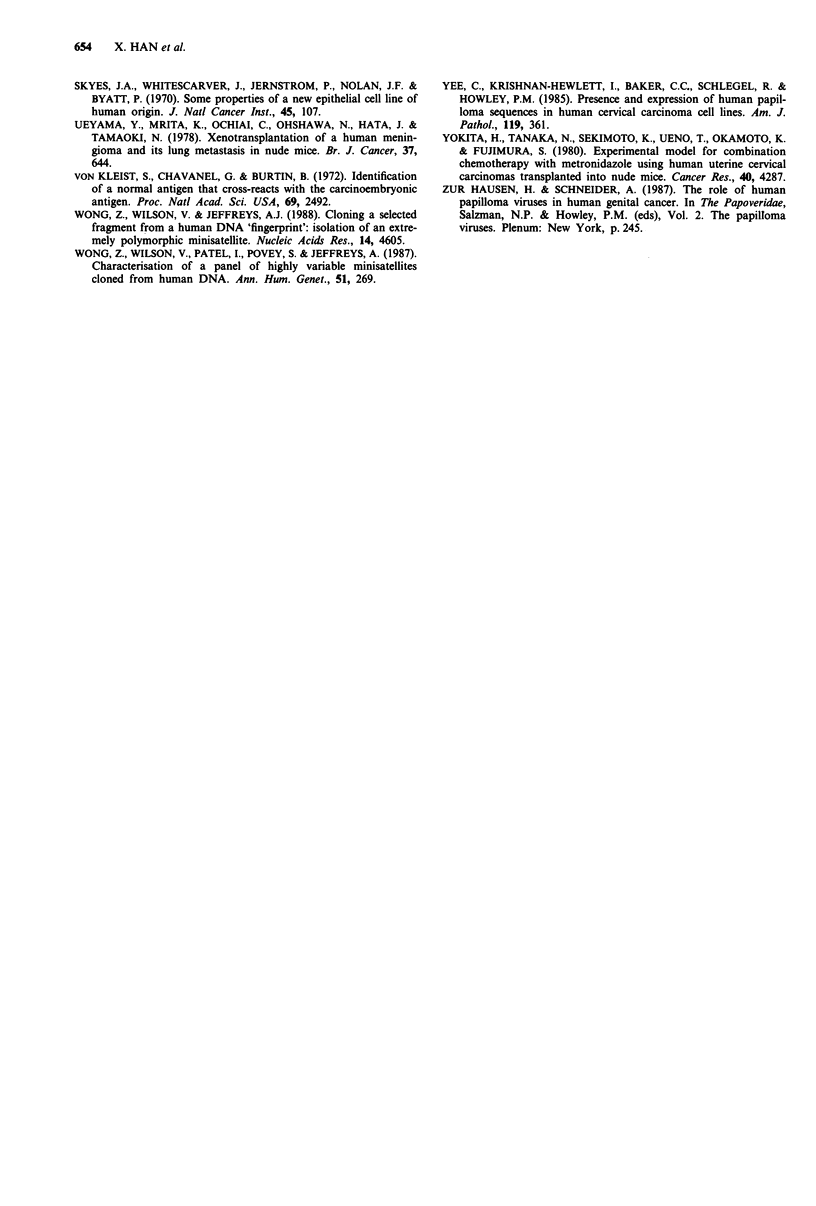# XH1--a new cervical carcinoma cell line and xenograft model of tumour invasion, 'metastasis' and regression.

**DOI:** 10.1038/bjc.1991.376

**Published:** 1991-10

**Authors:** X. Han, R. Lyle, D. L. Eustace, R. J. Jewers, J. M. Parrington, A. Das, T. Chana, B. Dagg, S. Money, T. D. Bates

**Affiliations:** Department of Histopathology, UMDS, St Thomas Hospital, London, UK.

## Abstract

**Images:**


Br. J. Cancer (1991), 64, 645-654 © Macmillan Press Ltd., 1991

### XH1 – a new cervical carcinoma cell line and xenograft model of tumour invasion, 'metastasis' and regression

X. Han[1], R. Lyle[1], D.L.S. Eustace[3], R.J. Jewers[2], J.M. Parrington[5], A. Das[1], T. Chana[1], B. Dagg[1], S. Money[1], T.D. Bates[4], A. Kenney[3] & E. Heyderman[1]

*Departments of [1]Histopathology, [2]Richard Dimbleby Laboratory of Cancer Virology, [3]Obstetrics and Gynaecology and [4]Radiotherapy, UMDS, St Thomas Hospital, London SE1 7EH, [5]MRC Human Biochemical Genetics Unit, University College, London NW1 2HE.*

**Summary** A new cell line, XH1, has been derived from an invasive focally keratinising adenosquamous carcinoma of the cervix in a 32 year old patient. It has been maintained in long term monolayer culture for 26 months, and passaged over 100 times (>>300 population doublings). It is aneuploid with a mean chromosome number of 78. Examination using two minisatellite hypervariable DNA probes has shown it to be different from other cell lines maintained in this laboratory and from HeLa. Two sublines, XH1a and XH1b, show marked differences in monolayer culture, growth in soft agar, and xenograft formation. XH1 and XH1a cells readily form subcutaneous xenografts, and lung colonies can be established by their intravenous injection. Subcutaneous injection of XH1b cells results in rapid cell growth for a few days after which the tumour undergoes degeneration and then regresses completely. The XH1 karyotype has many rearranged chromosomes. Parental XH1 cells and both sublines show integration of HPV16 into the genome.

In England and Wales 4% of cancer deaths are due to carcinoma of the cervix. There are approximately 4,000 new cases of invasive cervical carcinoma per annum, with an overall mortality of 50% (OPCS, 1990*a*). Whilst the total number of deaths has fallen slightly, there has been a significant increase in cervical cancer incidence and mortality in women under the age of 45. In 1974–1978 there were 692 deaths from cervical cancer in these younger women. In the next 5 years, 1979–1983, the number had increased to 986, with 1,203 deaths 1984–1988 (OPCS, 1977–1990*b*). During the same period deaths in older women fell from 9,607 in 1974–1978 to 8,595 in 1979–1984 to 7,860 in 1984–1988 (OPCS, 1977–1990[b]). A similar trend has been reported from elsewhere. In Australia, for example, the proportion of women under 35 with cervical cancer increased from under 9% of cases in the 1950's, to 25% in the 1970's and 1980's (Elliott *et al.*, 1989).

Squamous carcinoma is the commonest carcinoma of the cervix (Anderson, 1985), although if mucin stains are used, up to 30% are shown to be adenosquamous (Buckley & Fox, 1989). In most cases there appears to be a progression from mild cervical intra-epithelial neoplasia (CIN 1), through moderate (CIN 2), to severe dysplasia/carcinoma-*in situ* (CIN 3) before frank invasive carcinoma supervenes (Reagan *et al.*, 1953; Richart, 1967; Buckley *et al.*, 1982). In a small number of women, the diagnosis of invasive tumour is made soon after a previous negative smear, with no detectable transition stage (Ashley, 1966; Hakama & Penttinen, 1981; Peters *et al.*, 1988). The cytological characteristics of the cells involved in CIN 3 as well as those in invasive carcinoma are those of a malignant phenotype, and show pleomorphism, loss of polarity, abnormal mitotic figures and aneuploidy. The cells of invasive carcinoma have the ability to transgress the basement membrane, spread locally and metastasise, with a resulting 50% mortality (OPCS, 1990*b*). The cells of *in situ* carcinoma have not transgressed the basement membrane and may lack factors that would enable them to do so.

In order to study differences between normal, dysplastic and invasive malignant cervical epithelial cells we have established such cells in monolayer culture and as xenografts in nude mice. In this paper we report on a new cervical carcinoma cell line XH1, established during the course of this study, and on the cultural and xenograft behaviour of the two sublines, XH1a and XH1b, derived from it.

Correspondence: E. Heyderman, Department of Histopathology, UMDS, St Thomas Hospital, London SE1 7EH, UK.
Received 2 April 1990; and in revised form 10 May 1991.

### Materials and methods

### *Initiation of long term culture of XH1*

The XH1 cell line was established from a Wertheim's hysterectomy specimen of an adenosquamous carcinoma in a 32 year old patient. The tumour consisted mainly of moderately differentiated focally keratinising squamous carcinoma, with clear cell areas and foci of glandular differentiation containing PAS positive-diastase resistant mucin. Occasional multinucleate giant tumour cells were seen.

Minor modifications of methods previously published were used to establish the cell line (Stanley & Parkinson, 1979; Stanley & Dahlenburg, 1984). Portions of the tumour were minced and incubated in protease (Sigma Chemical Co, Dorset), collagenase (Lorne Diagnostics Ltd, Suffolk) and DNAase (Sigma ) in EBSS (Earle's Balanced Salt Solution) containing 100 IU penicillin $ml^{-1}$ and 100 $\mu g\,ml^{-1}$ streptomycin (Gibco BRL Ltd, Middx). Single cells and small clumps were seeded into flasks containing Mitomycin-C treated Swiss albino embryonic mouse fibroblast 3T3 cells (ATCC CCL92) (ICN Flow Laboratories Ltd, Bucks). An enriched medium was used consisting of DMEM (Dulbecco's Modification of Eagle's Medium) (ICN Flow) with 20% FCS, 2 mM L-glutamine, antibiotics, 0.4 $\mu g\,ml^{-1}$ hydrocortisone (Glaxo Laboratories Ltd, Middx), 10 $\mu g\,ml^{-1}$ bovine insulin (Sigma), 10 $ng\,ml^{-1}$ epidermal growth factor (ICN Flow), and 10 $ng\,ml^{-1}$ cholera toxin (Sigma).

At passage #4 a colony consisting predominantly of small rapidly dividing epithelial cells was selected and passaged further. Fibroblasts from the cervical stroma were removed by incubation with 0.02% EDTA in EBSS at 37°C for 2–3 min and then vigorous pipetting against the surface of the flask (Stanley & Parkinson, 1979). The 3T3 feeder layer was omitted from passage #6 onwards (>>300 population doublings).

A Mycotect kit (Gibco) was used to test for Mycoplasma infection at 3–4 week intervals.

### *XH1a and XH1b sublines*

At passage #29 XH1 single cells were plated in enriched medium at densities of 5–100 cells/well in 24-well plates on

Mitomycin-C treated 3T3 cells. Growth was only seen in wells seeded with 50–100 cells/well. After passage #4, 3T3 feeder cells were no longer required. Two sublines of cells emerged with differing cell morphologies (see Results). One of each type was selected, designated XH1a and XH1b. They were subjected to more than 80 further passages.

*Karyotyping*

Chromosome preparations from XH1 cells passage #6 were made by standard methods using colcemid for 1–2 h at a final concentration of 0.02 $\mu g\,ml^{-1}$ and hypotonic KCl/EDTA for 20 min (Seizinger *et al.*, 1987). Chromosomes from 30 metaphases were counted on aceto-orcein stained slides, and 13 cells from G-banded preparations were analysed in full from photographs.

*Hypervariable minisatellite DNA probing*

Locus-specific hypervariable minisatellite probes, MS1 and MS31 (ICI Biological Products, Cheshire) (Wong *et al.*, 1987; 1988) were used to ensure that the XH1 cells were not contaminated with the established cell lines Caski (Patillo *et al.*, 1977), A431 (Giard *et al.*, 1973) and Bowes melanoma (Rifkin *et al.*, 1974), also maintained in this laboratory.

DNA was isolated from the patient's blood and cultured cells by proteinase K (50 mg ml) (Boehringer Corporation London Ltd, BCL, Sussex) and phenol-chloroform extraction (Sambrook *et al.*, 1989). Ten $\mu$g DNA was digested with *Hinf*I (BCL) and electrophoresed in a 0.7% agarose gel until fragments smaller than 2 kb had run off the gel. After electrophoresis, the DNA fragments were denatured, transferred to Hybond-N (Amersham International plc, Bucks), and hybridised with MS31 labelled with digoxigenin by random hexanucleotide priming, according to the manufacturer's instructions (BCL) (Feinberg & Vogelstein, 1983). Though HeLa cells (Gey *et al.*, 1952) have not been grown in this laboratory, HeLa DNA (obtained from Dr Farrell, Ludwig Institute, London) was also probed to exclude contamination of the other cell lines with HeLa. Because the bands obtained using A431 and Caski ran in a similar position, DNA from these cell lines and from XH1 and Bowes melanoma was also probed with digoxigenin-labelled MS1.

*DNA preparation for HPV analysis*

Total cellular DNA was purified by lysing the cells in 10 mM Tris-Cl (pH 8) – 10 mM NaCl – 10 mM EDTA (pH 8) – 0.5% SDS containing 100 $\mu g\,ml^{-1}$ proteinase K and incubation overnight at 37°C. Proteins were removed by sequential extractions with equal volumes of phenol, phenol-chloroform-isoamyl alcohol (25:24:1) and chloroform-isoamyl alcohol (24:1). RNA was then removed by treatment with 50 $\mu g\,ml^{-1}$ RNAase A, followed by digestion with 100 $\mu g\,ml^{-1}$ proteinase K in 1% SDS and deproteination as above. DNA was precipitated with ethanol, resuspended in water and the concentration determined by spectrophotometry.

*PCR*

DNA amplification was performed using the degenerate primers MY09 (5'-CGTCC[A/C]A[A/G][A/G]GGA[A/T]ACT GATC-3') and MY11 (5'-GC[A/C]CAGGG[A/T]CATAA[C/T]AATGG-3') (Perkin-Elmer Cetus, ILS, London). These amplify an approximately 450 bp region located in the L1 open reading frame of all sequenced papillomaviruses. PCR reactions were performed in a volume of 100 $\mu$l in 10 mM Tris-Cl (pH 8.3) – 2.5 mM $MgCl_2$ – 50 mM KCl with deoxyribonucleotides at final concentrations of 200 $\mu$M each, primers at 1 $\mu$M and target DNA at 1 $\mu g\,ml^{-1}$. 2.5 U of *Taq* DNA polymerase (Perkin-Elmer Cetus) were added to this mixture and the whole overlaid with 100 $\mu$l mineral oil. The amplification mixture was incubated first at 94°C for 5 min then for 30 cycles of 94°C for 30 s, 50°C for 30 s and 72°C for 45 s each. Extension was completed by a final incubation at 72°C for 5 min.

Aliquots (one-tenth) of the amplification mixture were precipitated with ethanol, and each digested with *Bam*HI, *Eco*RI, *Hae*III, *Hinc*II or *Pst*I, and then analysed on 4% NuSieve-1% SeaKem agarose gels (FMC Products, Flowgen, Kent).

Human placental DNA was used as a negative control to confirm the absence of false positives in the PCR reactions.

*Hybridisation analysis*

Total cellular DNA in 10 $\mu$g aliquots either undigested or each digested with *Bam*HI, *Hind*III or *Pst*I, were each electrophoresed through 0.8% agarose gels in TAE buffer. After electrophoresis DNA samples were prepared and transferred to nylon membranes (HyBond-N, Amersham, Bucks) using a Posiblotter (Stratagene, NBL, Northumberland) under the conditions described by the manufacturers. A random primer labelling technique (MultiPrime, Amersham) was used to generate $^{32}$P-labelled HPV16 probes. These probes were used to challenge membrane bound DNA samples in 5 × SSC – 5 × Denhardts – 0.5% SDS – 100 $\mu g\,ml^{-1}$ denatured, sheared salmon sperm DNA at ($T_m$-20°C) for 16 h. They were washed first at low stringency ($T_m$-40°C) then at high stringency ($T_m$-10°C). Kilobase ladder DNA markers (BRL, Paisley) were used to determine the size of the PGR product.

*Culture in soft agar*

XH1a or XH1b cells were mixed with 0.5% agar solution (Agar Noble, Difco Laboratories, Surrey) in enriched medium to give a final agar concentration of 0.35%, and a cell density $5 \times 10^4\,ml^{-1}$. After 4 weeks the agar was decanted and colonies containing more than 50 cells were counted.

*Xenografts*

Xenograft production was attempted in 53 nude mice (athymic nu/nu on Balb/c background). Twelve were female, 41 were male and they ranged in age from 3–20 weeks. Single cell suspensions in PBS pH 7.2 of various passages of XH1, XH1a, XH1b cells, and a xenograft obtained by inoculation of passage #5 of XH1 cells, were used. Five × $10^6$ cells were injected subcutaneously into both pectoral regions of 43 mice, and $10^7$ cells were also injected intraperitoneally into three of these mice. 1–5 × $10^4$ cells were injected into the tail vein of 22 mice, 12 of which also had simultaneous bilateral subcutaneous inoculation. Eight mice died immediately after intravenous injection, and four more died unexpectedly. Mice bearing XH1 or XH1a xenografts were sacrificed either when the subcutaneous tumours reached 1.5–2 cm in maximum diameter, or seemed about to ulcerate. Mice bearing XH1b xenografts were sacrificed at 5 and 7 days and thereafter at weekly intervals up to 50 days.

*Immunocytochemistry*

Formalin-fixed paraffin-embedded sections of the primary tumour, pellets of XH1, XH1a and XH1b cells (Heyderman *et al.*, 1989), and sections of xenografts were stained with a variety of antibodies. Monoclonal antibodies to epithelial membrane antigen (EMA) (Heyderman *et al.*, 1979), carcinoembryonic antigen (CEA), low molecular weight cytokeratin (CAM 5.2 antibody), desmin and vimentin, and a rabbit polyclonal antibody to S100 were used (See Table I for sources and positive control tissues). They were also stained with TDM35 a murine monoclonal antibody raised against XH1 cells (Han *et al.*, 1990). An ABC method without enzyme predigestion (Hsu *et al.*, 1981) as employed using a biotinylated anti-species secondary antibody and streptavidin/biotin complex (Dako, Bucks). Endogenous peroxidase was inhibited by a sequence of hydrogen peroxide,

**Table I** Antibodies used, control tissues and sources

| *Antibody* | *Dilution* | *Control tissue* | *Source* |
|---|---|---|---|
| *Murine monoclonal antibodies* | | | |
| Epithelial membrane antigen (EMA) | 1:100 | 2° ovarian carcinoma | Dako, Bucks |
| Carcinoembryonic antigen (CEA) | 1:5 | colorectal carcinoma | Amersham, Bucks |
| Cytokeratin (CAM 5.2) | 1:4 | colorectal carcinoma | Becton Dickinson, Oxford |
| Vimentin | 1:20 | fibroid | Dako, Bucks |
| Desmin | 1:50 | striated muscle | Dako, Bucks |
| TDM35 | Neat | XH1 xenograft | Own laboratory |
| *Rabbit polyclonal antibody* | | | |
| S100 | 1:1000 | normal nerve | Dako, Bucks |

periodic acid, and potassium borohydride (Heyderman, 1986).

*Negative controls*

The relevant antigens for the antibodies used in this study were not available to perform specificity controls using antibody absorbed with the corresponding antigen (Heyderman *et al.*, 1989). However antibodies negative on the test sections would act as negative controls for the method and exclude false positivity due to endogenous peroxidase or to non-specific binding of the secondary antibodies or the ABC complex.

### Results

*Morphology*

Parental XH1 cells have been maintained in long term culture for 26 months, and passaged over 100 times. They grow as a flat adherent monolayer of polygonal epithelial cells, with occasional multinucleate giant cells. Some cells contain mucin, which is seen in small clumps in the medium just above them. XH1a cells have the same morphology in monolayer culture as the parental cells and grow in adherent sheets (Figure 1), while XH1b cells grow in a diffuse fashion until confluence (Figure 2). Some XH1b cells are flattened and hemi-spherical, while others are more spindle shaped. Both sublines have maintained their morphology over a further 80 passages. At EM level both XH1a and XH1b cells show occasional desmosomes and tonofilaments, confirming their squamous origin.

The cultures have always been free from Mycoplasma contamination.

*Karyotyping*

Karyotyping of XH1 cells at passage #6 showed them to be aneuploid, with a modal chromosome number of 78, and a

**Figure 1** Uncloned XH1 cells in monolayer culture showing mainly polygonal morphology with some giant cells (large arrow) and mucin-secreting cells (small arrow). XH1a cells have a very similar morphology, but giant cells are less frequent × 110.

**Figure 2** Until confluence XH1b cells show a non-cohesive pattern of growth, with retention of an epithelial morphology × 165.

range of 75–86 (Figure 3). There were a number of chromosome rearrangements whose probable origin could be determined. These were 4p +, 5q –, 10p +, 11p +, 11p –, 11q +, 12p –, 14p +, 14q +, and 15p +, and there were several markers of unknown origin (Figure 4). The 14p + probably contained material from 11q, and the 15p + material from 5q. Three of the rearrangements were present in duplicate, which suggests that they may have originated in a near-diploid cell which subsequently duplicated. A secondary mode of 82/83 had a slightly different karyotype in which chromosome 1 rearrangements were found. A detailed examination is being carried out of these differences and the karyotypes of XH1a and XH1b and clones derived from them. Full details will be reported elsewhere.

*MS31 Hypervariable minisatellite probing*

The MS31 probe showed similar bands with DNA extracted from the patient's blood after treatment and with that from XH1, XH1a and XH1b cells. The bands were different from HeLa (Figure 5), A431, Caski and Bowes melanoma (Figure 6). The bands from Caski and A431 ran in similar positions to each other though that from A431 appeared to show two

**Figure 3** Histogram showing frequency of chromosome number per metaphase in XH1 passage #6 (30 cells counted).

**Figure 4** Representative karyotype from XH1 passage #6 (m = marker of unknown origin).

**Figure 5** Southern blots of DNA from parental uncloned XH1, clone XH1a, clone XH1b, the patient's blood, and HeLa probed with minisatellite hypervariable MS31 labelled with digoxigenin.

**Figure 6** Southern blots of DNA from parental uncloned XH1, A431, CaSki and Bowes melanoma probed with MS31. Note the similarity between the A431 and CaSki bands, though A431 probably has two bands and CaSki only one with this probe.

bands and Caski one band (Figure 6). DNA from XH1, A431, Caski and Bowes melanoma was therefore challenged with another digoxigenin-labelled probe, MS1. This showed these three cell lines all to be of different origin (Figure 7).

*Analysis of PCR products and HPV hybridisation*

The PCR products from each of XH1, XH1a and XH1b were approximately 450 bp and exhibited the single *Eco*RI and *Pst*I sites characterstics of HPV16 (Seedorf *et al.*, 1985) (Figure 8). The fact that the amplimers recognised this sequence suggests that of the late genes at least the L1 region is conserved in the primary culture and sublines. With reference to the kilobase ladder DNA markers the identifiable bands were 451 bp for the undigested product, 234/217 bp for the *Eco*Rl digest and 216/205 bp for the *Pst*I

**Figure 7** Southern blots of DNA from parental uncloned XH1, A431, CaSki and Bowes melanoma probed with MS1. A431 and CaSki are clearly different from each other and from XH1 and Bowes melanoma.

**Figure 8** Restriction digests of PCR products. (1), (7) and (13) undigested PCR product; (2) (8) and (14) digested with *Bam*HI; (3), (9) and (15) *Eco*RI; (4), (10) and (16) *Hae*III; (5), (11) and (17) *Hinc*II; (6), (12) and (18) *Pst*I; (M) Kilobase ladder DNA markers: the identifiable marker bands are 1018, 516/505, 394, 344, 298, 220, 200, 154 and 142.

digest. The residual 30 bp fragment form the *Pst*I digest was not visible by these methods. HPV16 probed, Southern blotted total cellular DNA from XH1, XH1a and XH1b showed the presence of HPV16 sequences integrated monomerically into the host chromosomal DNA. Preliminary reconstruction experiments indicate that there are 50–100 copies per cell.

### *Growth characteristics*

The doubling time of XH1a in monolayer culture was 16.2 h and of XH1b 21.6 h. In soft agar XH1 cells grew in compact rounded colonies. XH1b cells formed diffuse clumps with loose contact between the cells. The mean colony forming efficiency (CFE) was 0.13% for XH1a cells and 0.08% for XH1b cells.

### *Xenograft results (Table II)*

*XH1 and XH1a* Nodules of subcutaneous tumour were first palpable 5–21 days after inoculation, and formed a mass 1.5 cm in diameter in 21–158 days. There was considerable

**Table II** Xenograft production

| | *Total mice* | *Died unexpectedly*[a] | *Subcutaneous tumours* | *Lung colonies* |
|---|---|---|---|---|
| *Uncloned* | | | | |
| SC[b] | 14 | 0 | 14/14 | – |
| SC[b] + IP[c] | 3 | 0 | 3/3 | – |
| SC[b] + IV | 3 | 0 | 3/3 | 1/3 |
| IV | 5 | 2 | – | 0/3 |
| Total | 25 | 2 | 20/20 (100%) | 1/6 (17%) |
| *XH1a* | | | | |
| SC[b] | 5 | 1 | 4/4 | – |
| SC[b] + IV | 7 | 3 | 4/4 | 2/4 |
| IV | 5 | 4 | – | 1/1 |
| Total | 17 | 8 | 8/8 (100%) | 3/5 (60%) |
| *XH1b*[d] | | | | |
| SC[b] | 9 | 2 | 6/7 (86%) | – |
| SC + IV | 2 | 0 | 2/2 (100%) | 0/2 |
| Total | 53 | 12 | 36/37 (97%) | 4/13 (31%) |

SC – subcutaneous inoculation. IP – Intraperitoneal inoculation. IV – Intravenous inoculation. [a]Eight mice died immediately after intravenous injection of XH1 cells. Four others were found dead unexpectedly. [b]All animals inoculated subcutaneously received bilateral injections. The results were regarded as positive if one or both gave rise to tumours. [c]All three mice injected intraperitoneally developed intra-abdominal xenografts. [d]Some XH1b tumours had completely regressed by the time the mouse was sacrificed.

variation in the growth rate and size of the tumour xenografts. The time the tumours took to reach 1.5 cm was independent of the passage number, or the sex or age of the mice.

Tumours derived from XH1 and XH1a cells showed an adenosquamous morphology similar to that of the original cervical carcinoma (Figures 9 and 10). Desmosomes and tonofilaments were seen at EM level (Figure 11 and 12). There was a variable amount of central necrosis, present sometimes in tumours less than 1 cm in maximum diameter as well as in some of the larger xenografts. There was focal glandular differentiation, and as in the primary tumour, epithelial mucin was demonstrable. The xenografts could be passaged from one mouse to another by inoculation of fresh fragments, and the passaged xenografts continued to show the same morphology. In the three mice inoculated intraperitoneally and subcutaneously, tumour was seen at both sites.

### *XH1b xenografts*

XH1b cells produced invasive tumours up to 6 mm in diameter within 6–8 days. Sections taken at this time showed them to consist of small sheets of poorly differentiated squamous carcinoma, surrounded by reactive macrophages and chronic inflammatory cells. In the centres of the sheets

**Figure 9** H&E preparation of the original adenosquamous carcinoma from which the XH1 cell line was derived. There is central keratinisation and some clear cell differentiation × 200.

**Figure 10** Subcutaneous XH1 xenograft showing blood vessel invasion × 200.

**Figure 11** Electron micrograph of XH1 xenograft showing abundant desmosomes.

**Figure 12** Elsewhere in the XH1 xenograft there were numerous tonofilaments.

the cells were seen to be degenerating and pyknotic (Figure 16). In nodules removed after 10–12 days, the tumours were seen to have regressed completely or undergone cystic degeneration, with the cyst wall consisting of loose vascular fibrous tissue. No tumour cells could be identified on H&E or immunostains. After 28 days no tumour was palpable and sections of the site showed cellular connective tissue and confirmed the absence of tumour.

*Local invasion*

Many of the subcutaneous xenografts showed invasion into host muscle, blood vessels (Figure 10), and perineural spaces.

*Lung colonisation*

Multiple small tumour deposits in the lung parenchyma were seen in the lungs of 4/11 mice 23–50 days after intravenous inoculation with XH1 (1/6) and XH1a cells (3/5) (Figure 13). Elsewhere in the lungs there were small fibrous nodules and calcified plaques in vessels that we interpret as the site of arrest of other tumour cells that did not become established (Figure 14). All of the mice had full histological examination of the lungs. None of the other mice that had not received intravenous injection of cells or with no evidence of lung colonisation showed similar nodules. Eight other mice died immediately following the intravenous injection of XH1 cells, and sections of the heart and lungs of six of them showed that injected tumour cells had reached the right ventricle and small pulmonary vessels, and had reaggregated into small clumps. Intravenous inoculation of XH1b cells did not result in the formation of lung colonies.

*Immunocytochemistry*

The original cervical carcinoma, XH1 and XH1a pellets and xenografts were positive in a strong but patchy fashion for cytokeratin (Figure 15) EMA, and CEA. XH1b xenografts and pellets showed patchy positivity for cytokeratin, EMA (Figure 16), and vimentin, but were negative for CEA. There was variable mainly weak co-expression of vimentin in the tumours, with stronger focal vimentin positivity in the cell pellets (Figure 17). The original tumour and all XH1 and XH1a xenografts and cell pellets showed strong positivity for TDM35 with the tumours positive mainly at the centre of nodules (Figure 18). XH1b cell pellets and xenografts were negative for TDM35. All of the tumours and cell pellets were

**Figure 13** Small nodule of tumour in mouse lungs following intravenous injection of XH1a cells × 200.

**Figure 14** Intravascular endothelialised fibrous nodule in mouse lungs following intravenous injection of XH1a cells. This probably represents the site of arrest of tumour cells which failed to become established × 200.

**Figure 15** XH1 xenograft shows patchy immunoperoxidase positivity for cytokeratin (CAM 5.2) × 200.

**Figure 18** XH1 xenograft immunostained with monoclonal antibody TDM35. The distribution of staining is again focal × 200.

**Figure 16** XH1b xenograft immunostained with anti-EMA. The tumour has begun to regress and cells at the centre are pyknotic × 320.

**Figure 17** XH1b cell pellet showing some cells strongly positive for vimentin × 320.

negative for S100 and desmin. Perineural spread and invasion by xenografts into the host muscle was highlighted using antibodies to S100 and desmin, for which the tumours were themselves negative.

### Discussion

In this paper data on the establishment and characterisation of XH1, a new cervical carcinoma cell line derived from an adenosquamous carcinoma is presented. Two sublines XH1a and XH1b were obtained and shown to be of epithelial origin. XH1a has the adherent epithelial morphology of the original primary cultures, while until confluence XH1b grows in a dissociated fashion, with hemispherical or spindle-shaped cells. Subclones of the cervical cell lines C4-I and C4-II have also been shown to have different morphologies and behaviour *in vivo* as well as *in vitro* (Auersperg, 1969; Auersperg *et al.*, 1989). A similar divergence into different morphological types was produced in a murine bladder carcinoma cell line by changing the culture conditions (Boyer *et al.*, 1989).

It is important to exclude contamination of new cell lines with HeLa or other established cell lines (Fogh *et al.*, 1977*a*). The hypervariable minisatellite probes MS1 and MS31 were used to show that DNA from XH1 and its sublines was like that extracted from the patient's white blood cells, and different from DNA from A431, Caski, Bowes melanoma, HeLa and mouse fibroblast 3T3 cells.

The karyotype of cultured tumour cells may undergo considerable changes *in vitro* and there is some evidence of chromosomal instability in XH1. However, since the culture was examined quite early on at passage #6, it is likely that many of the markers found were present in the original tumour. XH1 has a karyotype similar to other cervical carcinomas with many rearranged chromosomes. The small metacentric chromosome thought to be a 5q- is similar to markers found in 75% of direct preparations from primary cervical carcinomas (Atkin *et al.*, 1990). The marker $M_3$ in XH1 may represent a similar rearrangement. No normal copies of chromosome 11 were found in XH1 indicating that several rearrangements have occurred in this chromosome. Since the HeLa tumour suppressor gene has been localised to the long arm of chromosome 11 (Misra & Srivatsan, 1989), *in vivo* changes at this region might result in loss of 11q sequences that could be important in tumourigenesis. The XH1 sublines are being cloned by single cell selection and the karyotype differences between them will be investigated.

Several squamous cervical carcinoma cell lines have previously been reported. Some were derived from primary cervical carcinomas, including HeLa, Cl, C4 I and II, C12, C33 I and II, OG, SiHa, SW756, SKG, SKG-1, SKG-2 and SKG-III, Yumoto strain, HX151c, 155c, 156c and 160c (Gey *et al.*, 1952; Auersperg & Hawryluk, 1962; Auersperg, 1964; Auersperg, 1969; Arata *et al.*, 1969; Friedl *et al.*, 1970; Freedman *et al.*, 1982; Nozawa *et al.*, 1978; Ishiwata *et al.*, 1978; Yokita *et al.*, 1980; Nozawa *et al.*, 1982; Nozawa *et al.*, 1983; Kelland *et al.*, 1987). Others were derived from secondary deposits. These include CaSki, ME-180, MS751, HT-3, EC50 and DoT (Sykes *et al.*, 1970; Fogh & Trempe, 1975; Patillo *et al.*, 1977; Hussa *et al.*, 1978; Porter *et al.*, 1978). The W12 cell line was established from a CIN 1 cervical lesion (Stanley *et al.*, 1989). C4 I and II were classified as squamous carcinomas but showed mucin production (Auersperg, 1969), and the SKG-2 cell line also contained PAS-positive diastase resistant material (Ishiwata et al., 1978). These cell lines should therefore be reclassified as adenosquamous (Buckley & Fox, 1989). Although originally considered an epidermoid carcinoma, the HeLa cell line was later shown to have been derived from a cervical adenocarcinoma (Jones et al., 1971).

Production of xenografts in nude mice using the cervical carcinoma cell lines C4 I, C33, HT-3, ME-180, MS751 and SW732 was reported in a large study, but no details of local invasion were given, and none metastasised spontaneously (Fogh et al., 1977b). Other subcutaneous cervical carcinoma xenografts produced in nude mice include the SW756 (Porter et al., 1978), SKG-1 and SKG-2 (Ishiwata et al., 1978; Nozawa et al., 1982), and the HX151c, HX155c, HX156c and HX160c cell lines (Kelland et al., 1987). None of these metastasised, and generally host invasion was not commented upon, though stromal invasion was noted in the HX cell lines (Kelland et al., 1987). Marked cachexia was seen in mice bearing Yumoto xenografts and 80% of them died within 3 months, but none of the tumours metastasised. These authors also produced Hela subcutaneous xenografts, some of which metastasised spontaneously to the lungs (Yokita et al., 1980). In an experiment on the human patient from whom the OG cell line was derived, inoculation of both OG and HeLa into the thigh at the time of tumour recurrence elsewhere resulted in initial growth and then the tumours regressed (Arata et al., 1969). Growth of a subcutaneous xenograft of W12 cells derived at a site prepared by previous implantation of a glass coverslip has also been described (Stanley et al., 1989). Local invasion did not occur (Stanley, Personal communication).

The XH1 and XH1a xenografts are good models of local invasion. XH1 and XH1a cells will colonise the lungs after intravenous injection, but although vascular invasion was seen, none of the subcutaneous tumours had metastasised spontaneously. As the xenografts often threaten to ulcerate the overlying skin when they are less than 1 cm in diameter, experiments have to be terminated at this stage, which may be too early for metastasis. We intend removing primary subcutaneous xenografts when they are 0.75 cm in diameter, by which time micrometastasis could have occurred. The mice could then be maintained long enough for these metastatic cells to become established and detectable (Ueyama et al., 1978).

The original cervical tumour from which XH1 was derived, xenografts and cell pellets of XH1, XH1a and XH1b were positive for EMA and low molecular weight cytokeratin (CAM 5.2), consistent with their epithelial origin. They were also focally positive for vimentin. Co-expression of vimentin has been reported in a variety of epithelial neoplasms and is no longer considered to imply mesenchymal origin (Dabbs & Geisinger, 1988; Raymond & Leong, 1989). The primary cervical carcinoma, XH1 and XH1a xenografts and cell pellets were all positive for CEA and TDM35, which appears to recognise an NCA-like/CEA-like molecule (Von Kleist, 1972; Han et al., 1990). The XH1b xenografts and pellets were negative for CEA and for TDM35. CEA is a member of the immunoglobulin supergene family, and thought to be involved in cell adhesion and thereby to increase metastatic potential (Hostetter et al., 1990). Absence of CEA and of the antigen recognised by TDM35 may be factors militating against continued XH1b xenograft growth. Evidence that not all cells that arrest in the pulmonary vasculature continue to grow was shown by the presence of fibrous focally calcified nodules in lungs containing tumour colonies elsewhere.

There is a strong association between cervical carcinoma and human papillomavirus (HPV) (Zur Hausen & Schneider, 1977). XH1 and its sublines contain integrated HPV16 as do the other cervical carcinoma cell lines SiHa, CaSki, HX151c, HX155c, HX156c and HX160c, while HeLa, ME180, MS751, C4 I and C4 II contain HPV18 DNA. CaSki may have integrated HPV18 as well as HPV16; C-33A and HT-3 have neither (Yee et al., 1985; Pater et al., 1985; Spence et al., 1988). As both XH1a and XH1b contain HPV16 DNA, integration of HPV DNA does not explain their different morphology or xenograft behaviour. It has been suggested that patients whose cervical tumours evidence HPV integration have a better prognosis than those with HPV-negative tumours (Riou et al., 1990). The patient from whom XH1 was derived died of the disease within a year of diagnosis.

The XH1b subline provides a promising model of tumour regression and/or non-T cell mediated rejection. Possible mechanisms to be investigated by us include expression of the normal rather than a mutant p53 gene (Lane & Benchimol, 1990), lack of appropriate angiogenesis factors (Folkman & Klagsbrun, 1987), absence of plasminogen activators (Larsson et al., 1987) or of other proteases such as collagenase IV (Liotta et al., 1980). Nude mice are T cell but not natural killer (NK) cell deficient and possible increased susceptibility of XH1b cells to NK cells will also be examined (Richie, 1984).

We would like to thank Mr P.J. Warren and Mr M Groves for technical assistance with the early part of this study and the rest of the technical staff of the Department of Histopathology for valuable help with the preparation of blocks and H&E sections. We should also like to thank Dr M. Stanley for most useful advice on tissue culture, and Ms P. Chana for general laboratory assistance during her summer vacation. HeLa DNA was a generous gift from Dr Farrell, Ludwig Institute, St Mary's Hospital, London. The MS1 and MS31 probes were kindly donated by ICI Biological Products Ltd, Cheshire. The work was supported by St Thomas Hospital Research Endowments Fund, the Dunhill Medical Trust, the Jean Shanks Foundation, a National Westminister Bank grant for Cancer Research, and the Department of Health Procurement Directorate, as part of an antibody evaluation scheme.

### References

ANDERSON, M.C. (1985). The pathology of cervical cancer. *Clinics Obstet. Gynecol.*, **12**, 87.

ARATA, T., OGAWA, I., TANAKA, Y. & HASHIMOTO, K. (1969). Transplantation of a newly established human cancer cell lines. *Gann*, **60**, 649.

ASHLEY, J.L.B. (1966). Evidence for the existence of two forms of cervical carcinomas. *J. Obstet. Gynaec. Br. Cwlth.*, **73**, 382.

ATKIN, N.B., BAKER, M.C. & FOX, M.F. (1990). Chromosome changes in 43 carcinomas of the cervix uteri. *Cancer Genet. Cytogenet.*, **44**, 229.

AUERSPERG, N. (1964). Long-term cultivation of hypodiploid human tumor cells. *J. Natl Cancer Inst.*, **32**, 135.

AUERSPERG, N. (1969). Histogenetic behaviour of tumors. I. Morphologic variation *in vitro* and *in vivo* of two related human carcinoma cell lines. *J. Natl Cancer Inst.*, **43**, 151.

AUERSPERG, N. & HAWRYLUK, A.P. (1962). Chromosome observations on three epithelial cell cultures derived from carcinomas of the human cervix. *J. Natl Cancer Inst.*, **28**, 605.

AUERSPERG, N., KRUK, P.A., MACLAREN, I.A., WATT, P.M. & MYRDAL, S.E. (1989). Heterogeneous expression of keratin, involucrin, and extracellular matrix among subpopulations of a poorly differentiated human cervical carcinoma: possible relationships to patterns of invasion. *Cancer Res.*, **49**, 3007.

BOYER, B., TUCKER, G.C., VALLES, A.M., FRANKE, W.W. & THIERY, J.P. (1989). Rearrangements of desmosomal and cytoskeletal proteins during transition from epithelial to fibroblastoid organization in cultured rat bladder carcinoma cells. *J. Cell. Biol.*, **109**, 1495.

BUCKLEY, C.H. & FOX, H. (1989). Carcinoma of the cervix. In *Recent Advances in Histopathology* No 14. Anthony, P.P. & MacSween, R.N.M. (eds). Churchill Livingstone: Edinburgh, New York. Chapter 4; 63.

BUCKLEY, C.H., BUTLER, E.B. & FOX, H. (1982). Cervical intraepithelial neoplasia. *J. Clin. Pathol.*, **35**, 1.

DABBS, D.J. & GEISINGER, K.R. (1988). Common epithelial ovarian tumors. Immunohistochemical intermediate filament profiles. *Cancer*, **62**, 368.

ELLIOTT, P.M., TATTERSALL, M.H.N., COPPLESON, M. & 7 others (1989). Changing character of cervical cancer in young women. *Br. Med. J.*, **298**, 288.

FEINBERG, A.P. & VOGELSTEIN, B. (1983). A technique for radiolabelling DNA restriction endonuclease fragments to high specific activity. *Anal. Biochem.*, **132**, 6.

FOGH, J., FOGH, J.M. & ORFEO, T. (1977*b*). One hundred and twenty-seven cultured human tumor cell lines producing tumors in nude mice. *J. Natl Cancer Inst.*, **59**, 221.

FOGH, J. & TREMPE, G. (1975). New human tumour cell lines. In *Human Tumour Cells in vitro*. Fogh, J. (ed.), Plenum Press: New York, London, p.115.

FOGH, J., WRIGHT, W.C. & LOVELESS, J.D. (1977*a*). Absence of HeLa cell contamination in 169 cell lines derived from human tumors. *J. Natl Cancer Inst.*, **58**, 209.

FOLKMAN, J. & KLAGSBRUN, M. (1987). Angiogenic factors. *Science*, **235**, 442.

FREEDMAN, R.S., BOWEN, J.M., LEIBOVITZ, A. & 4 others (1982). Characterization of a cell line (SW756) derived from a human squamous carcinoma of the uterine cervix. *In Vitro*, **18**, 719.

FRIEDL, F., KIMURA, I., OSATO, T. & ITO, Y. (1970). Studies on a new human cell line (SiHa) derived from carcinoma of uterus. I, Its establishment and morphology (35091). *Proc. Soc. Exp. Med.*, **135**, 543.

GEY, G.O., COFFMAN, W.D. & KUBICEK, M.T. (1952). Tissue culture studies of the proliferative capacity of cervical carcinoma and normal epithelium. *Cancer Res.*, **12**, 264.

GIARD, D.J., AARONSON, S.A., TODARO, G.J. & 4 others (1973). *In vitro* cultivation of human tumors: establishment of cell lines derived from a series of solid tumors. *J. Natl Cancer Inst.*, **51**, 1417.

HAKAMA, M. & PENTTINEN, J. (1981). Epidemiological evidence for two components of cervical cancer. *Br. J. Obstet. Gynaecol.*, **88**, 209.

HAN, X., WARREN, P.J., LYLE, R., CHANA, T., BREMNER, F. & HEYDERMAN, E. (1990). XH1 – a new cervical cell line: xenograft and monoclonal antibody production. *J. Pathol.*, **160**, 155a.

HEYDERMAN, E. (1986). Tumour markers, In *Immunocytochemistry: Practical Applications in Pathology and Biology*, Polak, J.M. & Van Noorden, S. (eds), John Wright and Sons Ltd: Bristol, p. 502.

HEYDERMAN, E., EBBS, S.R., LARKIN, S.E., BROWN, B.M.E., HAINES, A.M.R. & BATES, T. (1989). Response of breast carcinoma to endocrine therapy predicted using immunostained pelleted fine needle aspirates. *Br. J. Cancer*, **60**, 630.

HEYDERMAN, E., STEELE, K. & ORMEROD, M.G. (1979). A new antigen on the epithelial membrane: its immunoperoxidase localisation in normal and neoplastic tissue. *J. Clin. Pathol.*, **32**, 35.

HEYDERMAN, E., WARREN, P.J. & HAINES, A.M.R. (1989). Commentary – Immunocytochemistry – problems and practice. *Histopathology*, **15**, 653.

HOSTETTER, R.B., CAMPBELL, D.E., KEREKHOFF, S. & 4 others (1990). Carcinoembryonic antigen enhances metastatic potential of human colorectal carcinoma. *Arch. Surg.*, **124**, 300.

HSU, S.-M., RAINE, L. & FANGER, H. (1981). Use of avidin-biotin-peroxidase complex (ABC) in immunoperoxidase techniques: a comparison between ABC and unlabelled antibody (PAP) procedures. *J. Histochem. Cytochem.*, **29**, 577.

HUSSA, R.O., PATILLO, R.A., RUCKERT, A.C.F. & SCHEUERMANN, K.W. (1978). Effects of butyrate and dibutyryl cyclic AMP on hCG-secreting trophoblastic and non-trophoblastic cells. *J. Endocrinol. Metab.*, **46**, 69.

ISHIWATA, I., NOZAWA, S., KIGUCHI, K., KURIHARA, S. & OKUMURA, H. (1978). Establishment of human uterine cervical cancer cell line and comparative studies between normal and malignant uterine cervical cells *in vitro*. *Acta. Obst. Gynaec. Jpn.*, **30**, 731.

JONES, H.W., MCKUSIK, V.A., HARPER, P.S. & KUANG-DONG, W. (1971). The HeLa cell and a reappraisal of its origin. *Obstet. Gynecol.*, **38**, 945.

KELLAND, L.R., BURGESS, L. & STEEL, G.G. (1987). Characterization of four new cell lines derived from human squamous carcinomas of the uterine cervix. *Cancer Res.*, **47**, 4947.

LANE, D.P. & BENCHIMOL, B. (1990). p53: oncogene or anti-oncogene. *Genes & Development*, **4**, 1.

LARSSON, G., LARSSON, A. & ASTEDT, B. (1987). Tissue plasminogen activator and urokinase in normal, dysplastic and cancerous squamous epithelium of the uterine cervix. *Thrombosis & Haemostasis*, **58**, 822.

LIOTTA, LA, TRYGGVASON, K., GARBISA, S., HART, I., FOLTZ, C.M. & SHAFIE, S. (1980). Metastatic potential correlates with enzymatic degradation of basement membrane collagen. *Nature*, **284**, 67.

MISRA, B.C. & SRIVATSAN, E.S. (1989). Localisation of HeLa cell tumor-suppressor gene to the long arm of chromosome 11. *Am. J. Hum. Genet.*, **45**, 565.

NOZAWA, S., IZUMI, S., OHTA, H. & 5 others (1978). On alkaline phosphatase of newly established cervical cancer cell line. *Acta. Histochem. Cytochem.*, **11**, 118.

NOZAWA, S., TSUKAZAKI, K., UDAGAWA, Y. & 4 others (1982). Human cell line (SKG-1) derived from epidermoid carcinoma of the cervix. In *Carcinoma of the Cervix: Biology and Diagnosis*. Hafez, E.S.E. & Smith, J.P. (eds), Martinus Nijhoff Publishers, The Hague/Boston/London. p. 142.

NOZAWA, S., UDAGAWA, Y., OHTA, H., KURIHAWA, S. & FISHMAN, W.H. (1983). Newly established uterine cervical carcinoma cell line (SKG-III) with Regan isoenzyme, human chorionic gonadotrophin $\beta$-subunit, and pregnancy-specific B1-glycoprotein phenotypes. *Cancer Res.*, **43**, 1748.

OFFICE OF POPULATION CENSUSES AND SURVEYS (1990*a*). *Cancer Statistics: Registrations*. Series MB1, No. 17, HMSO, London.

OFFICE OF POPULATION CENSUSES AND SURVEYS (1990*b*). *Mortality Statistics*. Cause series, DH2, N0. 15, HMSO, London, and Nos 1–14.

PATER, M.M. & PATER, A. (1985). Human papilloma virus types 16 and 18 sequences in carcinoma cell lines of the cervix. *Virology*, **145**, 313.

PATILLO, R.A., HUSSA, R.O., STORY, M.T., RUCKERT, A.C.F., SHALABY, M.R. & MATTINGLY, R.F. (1977). Tumor antigen and human chorionic gonadotropin in CaSki cells: a new epidermoid cervical cancer cell line. *Science*, **196**, 1456.

PETERS, R.K., THOMAS, D., SKULTIN, G. & HENDERSON, B.E. (1988). Invasive squamous carcinoma of the cervix after recent negative cytologic test results – a distinct subgroup? *Am. J. Obstet. Gynecol.*, **158**, 926.

PORTER, J.C., NALICK, R.H., VELLIOS, F., NEAVES, W.B. & MACDONALD, P.C. (1978). New tissue culture cell lines derived from human squamous cell carcinoma of the cervix and vagina. Squamous cells in tissue culture. *Am. J. Obstet. Gynecol.*, **130**, 487.

RAYMOND, W.A. & LEONG, A.S.-Y. (1989). Co-expression of cytokeratin and vimentin intermediate filaments in benign and neoplastic breast epithelium. *J. Pathol.*, **157**, 299.

REAGAN, J.W., SEIDEMAN, I.L. & SARACUSA, Y. (1953). The cellular morphology of carcinoma *in situ* and dysplasia or atypical hyperplasia of the uterine cervix. *Cancer*, **6**, 224.

RICHART, R.M. (1967). Natural history of cervical intraepithelial neoplasia. *Clin. Obstet. Gynecol.*, **10**, 748.

RICHIE, J.P. (1984). Abrogation of hematogenous metastasis in a murine model by natural killer cells. *Surgery*, **96**, 133.

RIFKIN, D.B., LOEB, J.N., MOORE, G. & RIECH, E. (1974). Properties of plasminogen activators formed by neoplastic human cell cultures. *J. Exp. Med.*, **139**, 1317.

RIOU, G., FAVRE, M., JEANNEL, D., BOURHIS, J., LE DOUSSAI, V. & ORTH, G. (1990). Association between poor prognosis in early-stage invasive cervical carcinomas and non-detection of HPV DNA. *Lancet*, **335**, 1171.

SAMBROOK, J., FRITSCH, E.F. & MANIATIS, T. (1989). *Molecular Cloning. A Laboratory Manual*. Cold Spring Harbor Laboratory, New York.

SEEDORF, K., KRAMMER, G., DURST, M., SUHAI, S. & ROWEKAMP, W.G. (1985). Human papillomavirus type 16 DNA sequence. *Virology*, **145**, 181.

SEIZINGER, B.R., DE LA MONTE, S., ATKINS, L., GUSELLA, J.F. & MARTUZA, R.L. (1987). Molecular genetic approach to human meningioma. Loss of genes on chromosome 22. *Proc. Natl Acad. Sci. USA*, **84**, 5419.

SPENCE, R.P., MURRAY, A., BANKS, L., KELLAND, L.R. & CRAWFORD, L. (1988). Analysis of human papillomavirus sequences in cell lines recently derived from cervical cancers. *Cancer Res.*, **48**, 324.

STANLEY, M.A., BROWNE, H.M., APPLEBY, M. & MINSON, A.C. (1989). Properties of a non-tumorigenic human cervical keratinocyte cell line. *Int. J. Cancer*, **43**, 672.

STANLEY, M.A. & DAHLENBURG, K. (1984). The effect of cholera-gen and epidermal growth factor on proliferation and maturation *in vitro* on human ectocervical cells. *In Vitro*, **20**, 144.

STANLEY, M.A. & PARKINSON, E.K. (1979). Growth requirements of human cervical epithelial cells in culture. *Int. J. Cancer*, **24**, 407.

SKYES, J.A., WHITESCARVER, J., JERNSTROM, P., NOLAN, J.F. & BYATT, P. (1970). Some properties of a new epithelial cell line of human origin. *J. Natl Cancer Inst.*, **45**, 107.

UEYAMA, Y., MRITA, K., OCHIAI, C., OHSHAWA, N., HATA, J. & TAMAOKI, N. (1978). Xenotransplantation of a human meningioma and its lung metastasis in nude mice. *Br. J. Cancer*, **37**, 644.

VON KLEIST, S., CHAVANEL, G. & BURTIN, B. (1972). Identification of a normal antigen that cross-reacts with the carcinoembryonic antigen. *Proc. Natl Acad. Sci. USA*, **69**, 2492.

WONG, Z., WILSON, V. & JEFFREYS, A.J. (1988). Cloning a selected fragment from a human DNA 'fingerprint': isolation of an extremely polymorphic minisatellite. *Nucleic Acids Res.*, **14**, 4605.

WONG, Z., WILSON, V., PATEL, I., POVEY, S. & JEFFREYS, A. (1987). Characterisation of a panel of highly variable minisatellites cloned from human DNA. *Ann. Hum. Genet.*, **51**, 269.

YEE, C., KRISHNAN-HEWLETT, I., BAKER, C.C., SCHLEGEL, R. & HOWLEY, P.M. (1985). Presence and expression of human papilloma sequences in human cervical carcinoma cell lines. *Am. J. Pathol.*, **119**, 361.

YOKITA, H., TANAKA, N., SEKIMOTO, K., UENO, T., OKAMOTO, K. & FUJIMURA, S. (1980). Experimental model for combination chemotherapy with metronidazole using human uterine cervical carcinomas transplanted into nude mice. *Cancer Res.*, **40**, 4287.

ZUR HAUSEN, H. & SCHNEIDER, A. (1987). The role of human papilloma viruses in human genital cancer. In *The Papoveridae*, Salzman, N.P. & Howley, P.M. (eds), Vol. 2. The papilloma viruses. Plenum: New York, p. 245.